# Exploring Test-Retest Reliability and Longitudinal Stability of Digital Biomarkers for Parkinson Disease in the m-Power Data Set: Cohort Study

**DOI:** 10.2196/26608

**Published:** 2021-09-13

**Authors:** Mehran Sahandi Far, Simon B Eickhoff, Maria Goni, Juergen Dukart

**Affiliations:** 1 Institute of Neuroscience and Medicine, Brain & Behaviour (INM-7) Research Centre Jülich Jülich Germany; 2 Institute of Systems Neuroscience, Medical Faculty Heinrich Heine University Düsseldorf Düsseldorf Germany

**Keywords:** health sciences, medical research, biomarkers, diagnostic markers, neurological disorders, Parkinson disease, mobile phone

## Abstract

**Background:**

Digital biomarkers (DB), as captured using sensors embedded in modern smart devices, are a promising technology for home-based sign and symptom monitoring in Parkinson disease (PD).

**Objective:**

Despite extensive application in recent studies, test-retest reliability and longitudinal stability of DB have not been well addressed in this context. We utilized the large-scale m-Power data set to establish the test-retest reliability and longitudinal stability of gait, balance, voice, and tapping tasks in an unsupervised and self-administered daily life setting in patients with PD and healthy controls (HC).

**Methods:**

Intraclass correlation coefficients were computed to estimate the test-retest reliability of features that also differentiate between patients with PD and healthy volunteers. In addition, we tested for longitudinal stability of DB measures in PD and HC, as well as for their sensitivity to PD medication effects.

**Results:**

Among the features differing between PD and HC, only a few tapping and voice features had good to excellent test-retest reliabilities and medium to large effect sizes. All other features performed poorly in this respect. Only a few features were sensitive to medication effects. The longitudinal analyses revealed significant alterations over time across a variety of features and in particular for the tapping task.

**Conclusions:**

These results indicate the need for further development of more standardized, sensitive, and reliable DB for application in self-administered remote studies in patients with PD. Motivational, learning, and other confounders may cause variations in performance that need to be considered in DB longitudinal applications.

## Introduction

Parkinson disease (PD) is primarily characterized by motor signs and symptoms, including tremor at rest, rigidity, akinesia, and postural instability [1]. Although standard in-clinic assessments such as the Unified Parkinson's Disease Rating Scale (UPDRS) are popular, they are influenced by interrater variability by relying on self-reporting by patients and caregivers or clinicians’ judgement [2]. In addition, they are costly and limited with respect to observation frequency.

The emergence of new technologies has led to a variety of sensors (ie, acceleration, gyroscope, GPS, etc) embedded in smart devices for daily use (ie, smartphone, smartwatch). Such sensor data, alongside other digital information recorded passively or when executing prespecified tasks, may provide valuable insight into health-related information. Such applications are now commonly referred to as digital biomarkers (DB) [3-5]. DB being collected frequently over a long period of time can provide an objective, ecologically valid, and more detailed understanding of the inter- and intra-individual variability in disease manifestation in daily life.

Numerous DB have been proposed for PD diagnosis as well as for assessing agreement between clinical rating scales such as UPDRS and sensor-driven data to quantify disease severity or intervention effects [4,6-9]. Despite these various proof of concept studies, many technical challenges with respect to DB deployment remain unaddressed. DB measures are prone to large variation caused by technical and procedural differences, including but not limited to placement/orientation, recording frequency of the devices, and environmental and individual variation (ie, due to motivation, medication, or other aspects) [10-12]. Other factors such as the effect of users' familiarity with technology and the impact of learning on the performance of measured DB in remote and self-administered PD assessment are other important sources of variation that have not been addressed so far. All of these factors may limit the sensitivity and reliability of DB measurements for any of the above PD clinical applications. DB longitudinal variation is therefore an important attribute that should be quantified and addressed. The reliability of DB assessment has been broadly studied for gait, balance, voice, and tapping data [13-18]. However, the existing studies typically focused on a single or a few aspects of PD, and most of them established the test-retest reliability in a standardized clinical setting, limiting the translatability of their findings to at-home applications. Among the studies that evaluated DB assessments for remote monitoring of PD, only one reported the test-retest reliability [4]. No PD studies systematically evaluated the test-retest reliability and longitudinal sensitivity of DB in a fully unsupervised and self-administered PD longitudinal setting.

Although various factors such as medication, disease severity, learning effects, bias from self-reporting, inconsistent disease severity, motivational impacts, and design protocols in self-administered studies can affect the long-term stability of DB, little attention has been paid to evaluating the reliability and longitudinal stability of DB in loosely controlled self-administered settings in daily life. Here, we aimed to address these open questions by assessing the test-retest reliability and longitudinal stability of gait, balance, speech, and tapping tasks in patients with PD and a control cohort consisting of healthy volunteers (HC) in an unsupervised and self-administered daily life setting using the large-scale m-Power data set [19].

## Methods

### Study Cohort

To address the open questions on the performance of DB measures in PD when collected in a self-administered setting in daily life, we first performed a comprehensive literature search identifying 773 DB features reported in previous studies to cover PD-related alterations in gait characteristics, tremor, postural instability, voice, and finger dexterity. We evaluated the longitudinal stability and test-retest reliability of these features as collected using 4 commonly applied PD tasks (gait, balance, voice, and tapping) in daily life using smartphone in a large cohort of self-reported patients with PD and healthy controls, the m-Power study [19-22]. In addition, we evaluated their sensitivity to learning and medication effects.

Enrolment in the m-Power study was open to adult participants who own an iPhone, are living in the United States, and are comfortable enough with English to read the instructions in the app. Participants were asked to download the app and complete a one-time demographic survey during registration. Demographic data include but are not limited to age, sex, health history, and previous PD clinical diagnosis. They also were asked to fill out a survey with selected questions from the UPDRS Section I (nonmotor experience) and Section II (motor experience), as well as the Parkinson’s Disease Questionnaire (PDQ-8). All the participants were suggested to complete each task (walking, tapping, voice, and memory) up to 3 times a day for up to 6 months. In addition, self-reported patients with PD were asked to complete the task before medication, after medication, and at another time when they were feeling at their best. 

Ethical oversight of the m-Power study was obtained from the Western Institutional Review Board. Prior to signing an electronically rendered traditional informed consent form, prospective participants had to pass a 5-question quiz evaluating their understanding of the study aims, participant rights, and data sharing options. After completing the e-consent process and electronically signing the informed consent form, participants were asked for an email address to which their signed consent form was sent and allowing for verification of their enrolment in the study. Participants were given the option to share their data only with the m-Power study team and partners (“share narrowly”) or to share their data more broadly with qualified researchers worldwide, and they had to make an active choice to complete the consent process (no default choice was presented). The data used in our study consist of all individuals who chose to have their data shared broadly.

### Data Preprocessing

**Figure 1:**
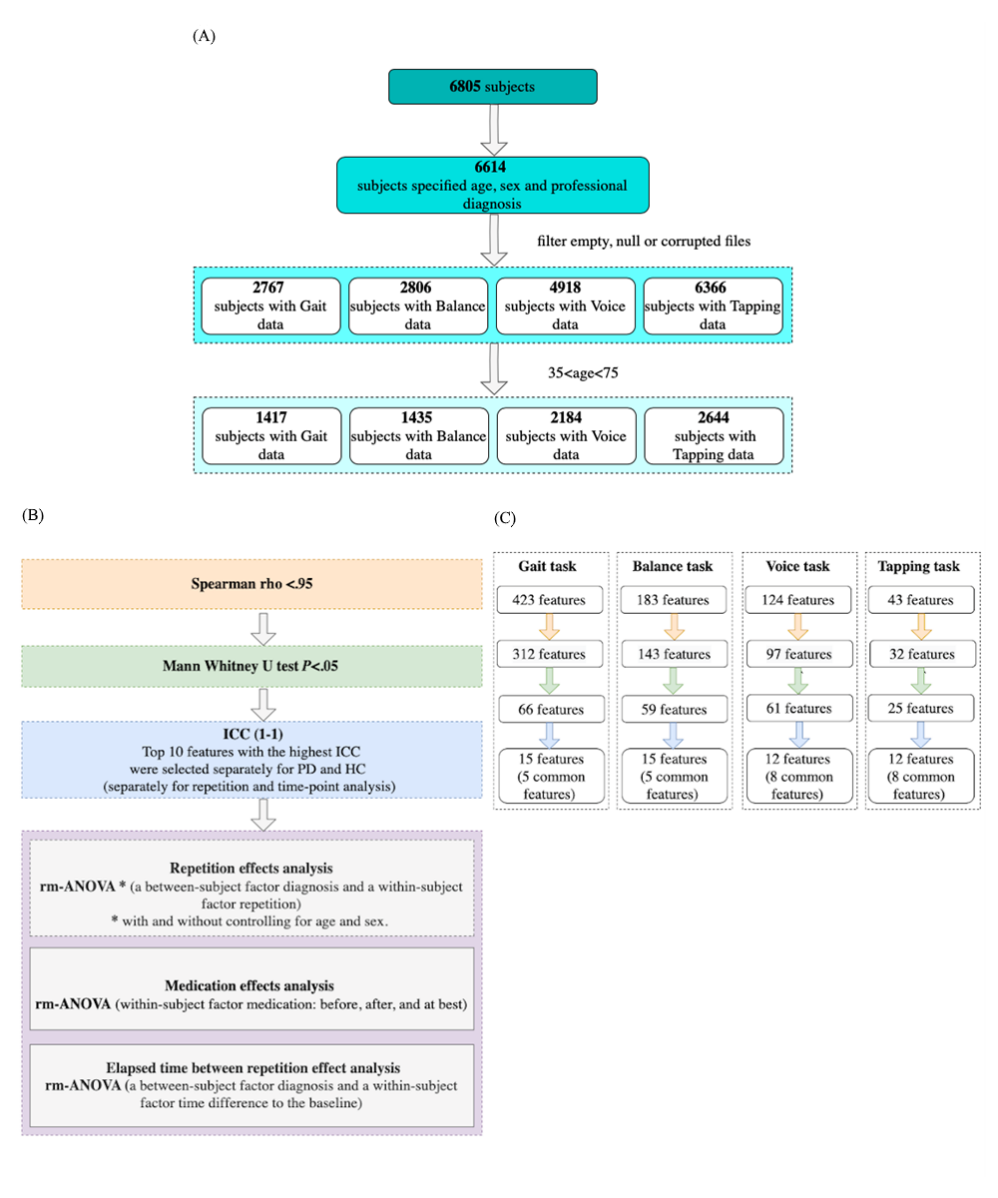
Overview of statistical analyses and the preprocessing scheme. (A) Flowchart of preprocessing steps. (B) Flowchart of statistical analyses. (C) Flowchart of number of features at each selection step. HC: healthy controls; ICC: intraclass correlation coefficients; PD: Parkinson disease; rm-ANOVA: repeated-measures analysis of variance.

The m-Power data set is assessed outside of a clinical environment with limited quality control and supervision. All information, including the health history, disease diagnosis, duration, treatment, and survey outcomes, are self-reported. To address these, we excluded participants who did not specify their age, sex, and information on professional diagnosis (if they belong to the PD or HC group) and those with empty, null, or corrupted files. The participants are assigned to the PD or HC group according to their response to the question “Have you been diagnosed by a medical professional with Parkinson disease?” There was a significant difference in the age and sex distribution between HC and PD groups. Particularly, age slanted toward younger and male individuals in HC. To reduce the impact of age, we restricted the age range for our analysis to between 35 and 75 years. The demographic details are provided in Table 1, and the overall overview of preprocessing steps is displayed in Figure 1A.

**Table 1:** Characteristics of study cohorts after data cleaning.

Characteristic			Gait					Balance					Voice					Tapping		
			HC^a^		PD^b^		HC			PD		HC			PD		HC			PD
**Sex,^c^ n**																				
	Male	655		399		668			401		1042			571		1370			630	
	Female	152		211		155			211		249			322		304			340	
Age (years),^c^ mean (SD)			49 (10.60)		60.3 (8.90)		48.9 (10.70)			60.3 (8.90)		47.7 (10.40)			60.1 (9)		46.9 (10.1)			59.9 (9)
UPDRS,^d^ mean (SD)			N/A^e^		12.60 (7.11)		N/A			12.53 (7.07)		N/A			12.58 (7.70)		N/A			12.54 (7.73)
UPDRS I, mean (SD)			N/A		4.90 (3.12)		N/A			4.9 (3.11)		N/A			4.93 (3.25)		N/A			4.95 (3.27)
UPDRS II, mean (SD)			N/A		7.76 (5.41)		N/A			7.7 (5.40)		N/A			7.61 (5.70)		N/A			7.56 (5.70)
PDQ-8,^f^ mean (SD)			N/A		5.13 (4.72)		N/A			7.07 (4.70)		N/A			5.28 (5.01)		N/A			5.3 (4.96)

^a^HC: healthy controls.

^b^PD: Parkinson disease.

^c^*P*<.001 (two-sample, two-tailed *t* test for age and chi-square test for sex with 95% confidence) for all tasks.

^d^UPDRS: Unified Parkinson's Disease Rating Scale.

^e^N/A: not applicable.

^f^PDQ: Parkinson’s Disease Questionnaire.

### Feature Extraction 

To identify features that are commonly used for the walking, voice, and tapping tasks for PD applications, we performed a comprehensive literature search in PubMed with the following terms: ((Parkinson's disease) AND (walking OR gait OR balance OR voice OR tapping) AND (wearables OR smartphones)). Based on this search, we identified a total of 773 features related to gait (N=423), balance (N=183), finger dexterity (N=43), and speech impairment (N=124). All of these features were computed for the m-Power study [23]. A detailed explanation of the extracted features, including the respective references, is provided in Tables S1-S4 in Multimedia Appendix 1. For features sharing the same variance (high pairwise correlation: Spearman ρ>0.95), only one of the features was selected randomly for further analyses to reduce the amount of redundant information for each task. Figure 1C summarizes the feature extraction process and the number of features at each selection step.

#### Gait and Balance

Impairments in gait speed, stride length, and stride time variability are common changes that are linked to PD [24-27]. Instability in postural balance is also considered to be one of the well-reported characteristics associated with PD [15,28-30]. Both were assessed by a walking task. The gait part consisted of 20 steps walking in a straight line, followed by the balance part of a 30-second stay still period. Given a heterogeneity of gait signal lengths across participants, we used a fixed length signal of 10 seconds and selected data from participants who met this criterion, which resulted in 28,150 records from 1417 unique participants. In addition to the accelerometer signals (x, y, and z), their average, the step series, position along the three axes by double integration, and velocity and acceleration along the path were used for feature extraction [31,32] (Table S1 in Multimedia Appendix 1). For balance, we used a 15-second time window, trimming the first 5 and the last 10 seconds of the 30-second records to reduce the noise due to the between-task transition period, resulting in 29,050 records from 1435 unique participants. Feature extraction covered signals related to tremor acceleration predicted to fall in the 4-7 Hz band and postural acceleration (nontremor) falling in the 0-3.5 Hz band [33] (Table S2 in Multimedia Appendix 1).

#### Voice

PD may also affect breathing and results in alterations in speech and voice. Reduced volume, hoarse quality, and vocal tremor are commonly reported for PD using voice analysis [16,34,35]. In this task, participants said “aaaah” for about 10 seconds. For voice, 49,676 records were selected, belonging to 2184 unique participants. Voice features were computed from fundamental frequency, amplitude, and period signals, trimming the first and the last 2 seconds of the 10-second interval (Table S3 in Multimedia Appendix 1).

#### Tapping

Impairment in finger dexterity is another sign associated with PD [36,37]. In the m-Power study, participants were asked to tap as fast as possible for 20 seconds with the index and middle fingers on the screen of their phone (positioned on a flat surface). Screen pixel coordinate (x, y) and timestamp of taped points plus acceleration sensor data were collected for this task. Overall, 55,894 recordings were selected, belonging to 2644 unique participants. Features were computed based on the intertapping distance and interval (Table S4 in Multimedia Appendix 1).

### Statistical Analysis 

For features to be considered usable for biomarker purposes in longitudinal studies, several criteria are important, including sensitivity to disease signs and symptoms, good test-retest reliability, and robustness against the effects of learning and other longitudinal confounders. To address these criteria, we adopted a stepwise statistical procedure (see Figure 1B for a summary of statistical analyses).

As DB measures are frequently not normally distributed, Mann-Whitney *U* tests were used to identify all features that significantly differ between PD and HC at the first administration (baseline) (*P*<.05). Effect sizes (Cohen *d*) were computed for these features to provide an estimate of the magnitude of differentiation between PD and HC.

Next, intraclass correlation coefficients (ICC, type 1-1) were used to determine the test-retest reliability of features showing a significant differentiation between PD and HC. We used ICC type 1-1 in our study because individuals were not tested under the same conditions (ie, same device), and reliability was determined from a single measurement. ICC values of 0-0.40 were considered to be poor, 0.40-0.59 to be fair, 0.60-0.74 to be good, and 0.75-1.00 to be excellent [38]. To assess the reliability of each feature, ICC values were computed for different time points versus baseline (one hour [0-6 hours], one day [calendric day], one week [7 calendric days], or one month apart [30 calendric days]), as well as for different repeats versus baseline (baseline vs second, third, fourth, and fifth repeat). We then focused our analyses on the top 10 features (as they provide a representative subset of the best performing features) with the highest median ICC values for each group (PD, HC) and tested for their longitudinal stability over time. Results for all features are reported in Multimedia Appendix 1. Features from the PD group are further referred to as “PD features,” those from the HC group only as “HC features,” and overlapping features from both groups as “common features.” We computed repeated-measures analyses of variance (rm-ANOVA) using a mixed factorial design with a between-subject factor diagnosis and a within-subject factor repetition (first, second, third, fourth, and fifth) including their interaction (Equation S1 in Multimedia Appendix 1). Participants who had at least 4 repetitions after baseline (463 for gait, 597 for balance, 1085 for voice, and 1333 for tapping) were included in these analyses. To assess the effects of age and sex on the longitudinal stability of the most reliable features, we repeated all analyses while controlling for age and sex as covariates (Equation S2 in Multimedia Appendix 1). Also, we assessed the impact of elapsed time between repetitions by computing rm-ANOVA using a mixed factorial design with a between-subject factor diagnosis and a within-subject factor elapsed time (calculated as a time difference of each repetition from the baseline in hours) and controlling for age and sex (Equation S3 in Multimedia Appendix 1).

Lastly, we assessed the impact of PD medication by computing rm-ANOVA in the PD group with the within-subject factor medication (ie, before, after, and at best) (Equation S4 in Multimedia Appendix 1). Participants with PD who had at least one marked task for each of the 3 PD medication conditions (ie, before, after, and at best) were included in treatment effect analysis (188 for gait, 189 for balance, 280 for voice, and 338 for tapping).

## Results

### Differentiation Between PD and HC

**Figure 2:**
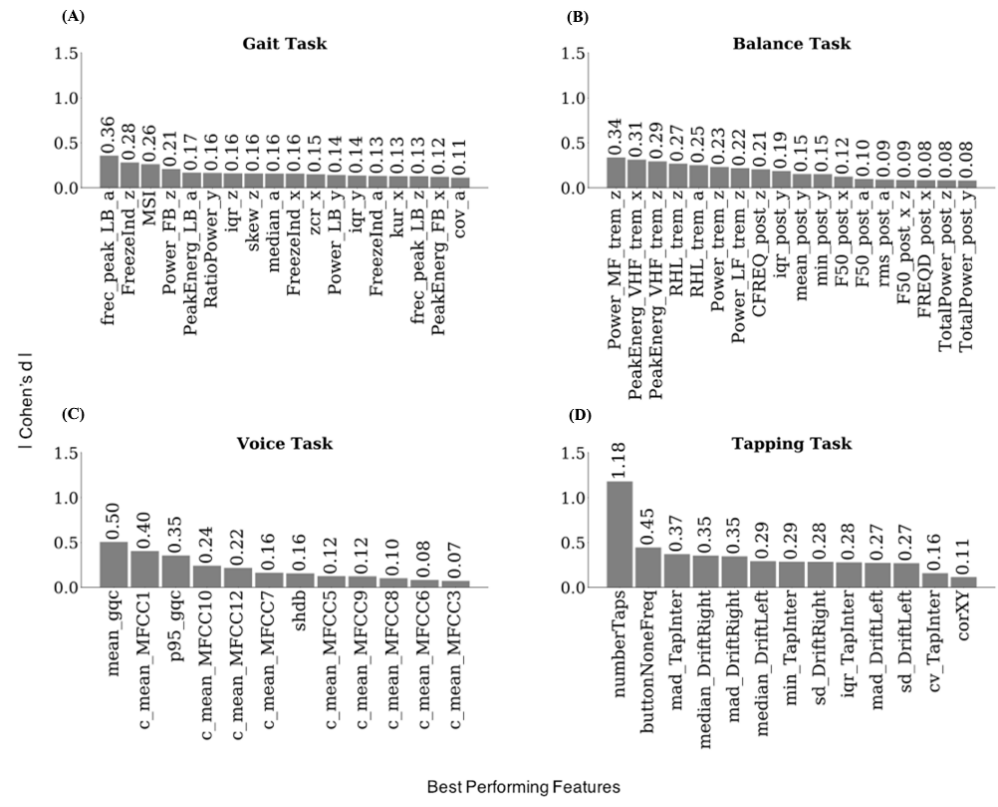
Effect size (Cohen d) for the most reliable features in the Parkinson disease and healthy control groups selected from different time points and repetitions. a: accelerometer average signal; iqr: interquartile range; min: minimum value; PeakEnerg: peak of energy; x: accelerometer mediolateral signal; y: accelerometer vertical signal; z: accelerometer anteroposterior signal. (A) Gait task. cov: coefficient of variation; FB: freezing band; frec_peak: frequency at the peak of energy; FreezeInd: freeze index; kur: kurtosis; LB: locomotor band; MSI: mean stride interval; RatioPower: sum of the power in the freezing and locomotor band; skew: skewness; zcr: zero-crossing rate. (B) Balance task. buttonNoneFreq: frequency of tapping outside the button; CFREQ: centroidal frequency; F50: frequency containing 50% of total power; FRQD: frequency of dispersion of the power spectrum; HF: high frequency (>4 Hz); LF: low frequency (0.15-3.5 Hz); MF: medium frequency (4-7 Hz); post: postural; Power: energy between 3.5-15 Hz; RHL: ratio between power in high frequency and low frequency; rms: root mean square; TotalPower: energy between 15-3.5 Hz; trem: tremor; VHF: very high frequency (>7 Hz). (C) Voice task. c_mean: mean of the MFCC; gqc: glottis quotient close; log: energy of the signal and the first and second derivatives of the MFCC; MFCC: Mel-frequency cepstral coefficients; p95: 95th percentile; shbd: shimmer. (D) Tapping task. corXY: correlation of X and Y positions; cv: coefficient; DriftLeft: left drift; DriftRight: right drift; mad: median absolute deviation; numberTaps: number of taps; sd: standard deviation; TapInter: tap interval.

First, we aimed to restrict the test-retest reliability analyses of the initial 773 features to those which significantly differ between PD (N=610 to 970 depending on the task, Table 1) and HC (N=807 to 1674). For this, we performed group comparisons for all computed features for gait, balance, voice, and tapping tasks. Overall, 66 out of 423 gait, 59 out of 183 balance, 60 out of 124 voice, and 25 out of 43 tapping features differed significantly (all *P*s<.05) between PD and HC at baseline (Figure 1C) with small (gait and balance) to medium effect sizes for gait, balance, and voice and small to large effect sizes for the tapping task (Figure 2 and Tables S5-S8 in Multimedia Appendix 1).

### Test-Retest Reliability

**Figure 3:**
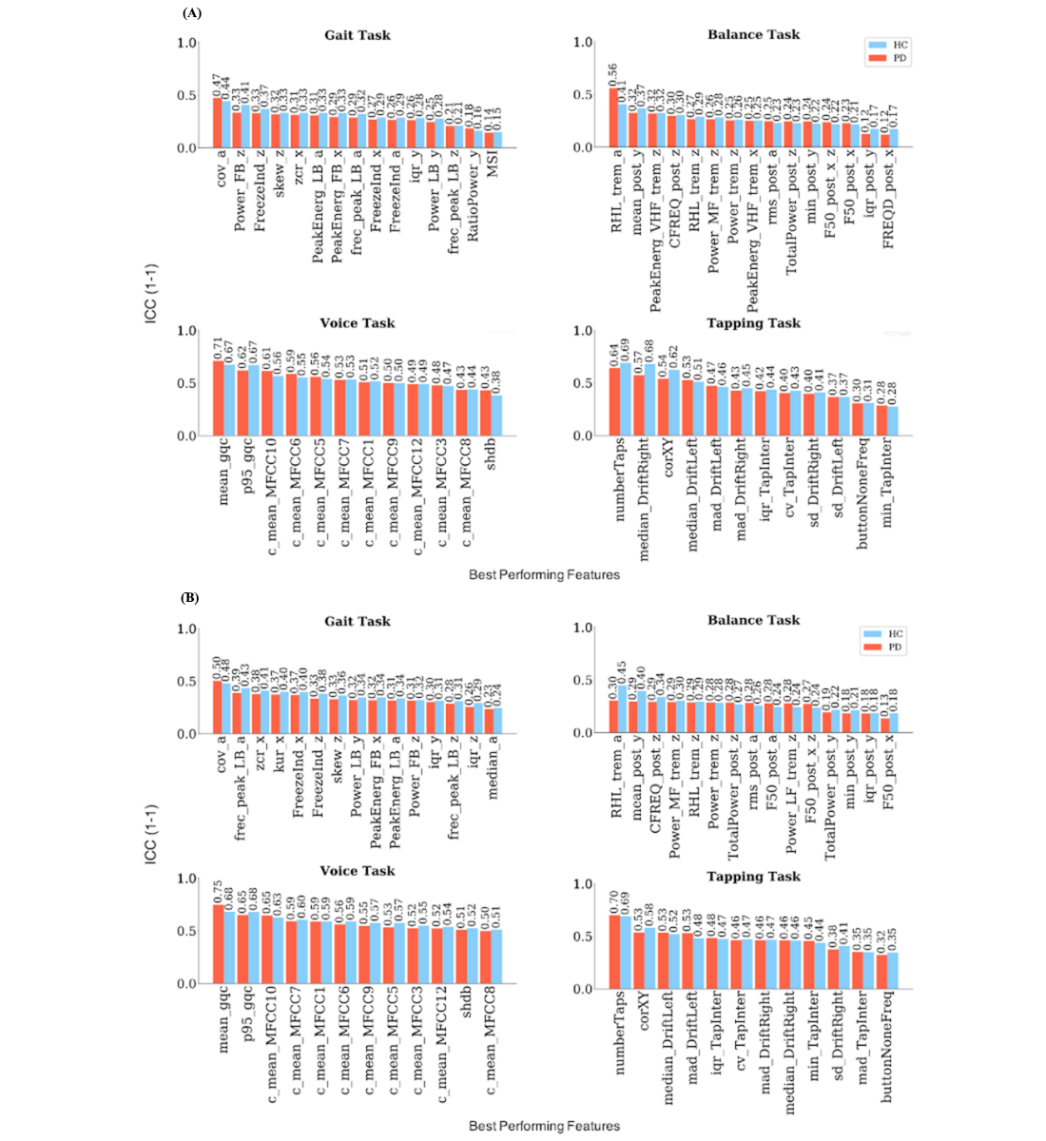
Median ICC values for the most reliable features in the Parkinson disease and healthy control groups. a: accelerometer average signal; ICC: intraclass correlation coefficient; iqr: interquartile range; min: minimum value; PeakEnerg: peak of energy; x: accelerometer mediolateral signal; y: accelerometer vertical signal; z: accelerometer anteroposterior signal. (A) Median ICC values across different time points for the best performing features. (B) Median ICC values across different repetitions for the best performing features. Gait task—cov: coefficient of variation; FB: freezing band; frec_peak: frequency at the peak of energy; FreezeInd: freeze index; kur: kurtosis; LB: locomotor band; MSI: mean stride interval; RatioPower: sum of the power in the freezing and locomotor band; skew: skewness; zcr: zero-crossing rate. Balance task—buttonNoneFreq: frequency of tapping outside the button; CFREQ: centroidal frequency; F50: frequency containing 50% of total power; FRQD: frequency of dispersion of the power spectrum; HF: high frequency (>4 Hz); LF: low frequency (0.15-3.5 Hz); MF: medium frequency (4-7 Hz); post: postural; Power: energy between 3.5-15 Hz; RHL: ratio between power in high frequency and low frequency; rms: root mean square; TotalPower: energy between 15-3.5 Hz; trem: tremor; VHF: very high frequency (>7 Hz). Voice task—c_mean: mean of the MFCC; gqc: glottis quotient close; log: energy of the signal and the first and second derivatives of the MFCC; MFCC: Mel-frequency cepstral coefficients; p95: 95th percentile; shbd: shimmer. Tapping task—corXY: correlation of X and Y positions; cv: coefficient; DriftLeft: left drift; DriftRight: right drift; mad: median absolute deviation; numberTaps: number of taps; sd: standard deviation; TapInter: tap interval.

Next, we identified the top 10 features with highest median test-retest reliability (as measured using ICC) separately for PD and HC across different time points (one hour, one day, one week, or one month apart) and repetitions (all participants with 5 repetitions of the task) (Tables S5-S8 in Multimedia Appendix 1, Figure 1B). This procedure resulted in 12 to 15 features (including shared ones) being selected for each task (Figure 3, Figures S1 and S2 in Multimedia Appendix 1). ICC analyses revealed poor to good test-retest reliability for these most reliable features from the gait and balance tasks and good to excellent reliability for features from voice and tapping tasks (Figure 3). The average ICC across the best performing features selected from different repetitions was lower at the fifth repetition compared to the first; it dropped from 0.11 to 0.09 for gait, from 0.21 to 0.13 for balance, from 0.39 to 0.24 for voice, and from 0.3 to 0.23 for tapping. The average ICC across the best performing features selected from different time points was also lower at one month compared to one hour apart, decreasing from 0.13 to 0.07 for gait, from 0.2 to 0.12 for balance, from 0.33 to 0.26 for voice, and from 0.32 to 0.19 for tapping.

### Repetition Effects

**Figure 4:**
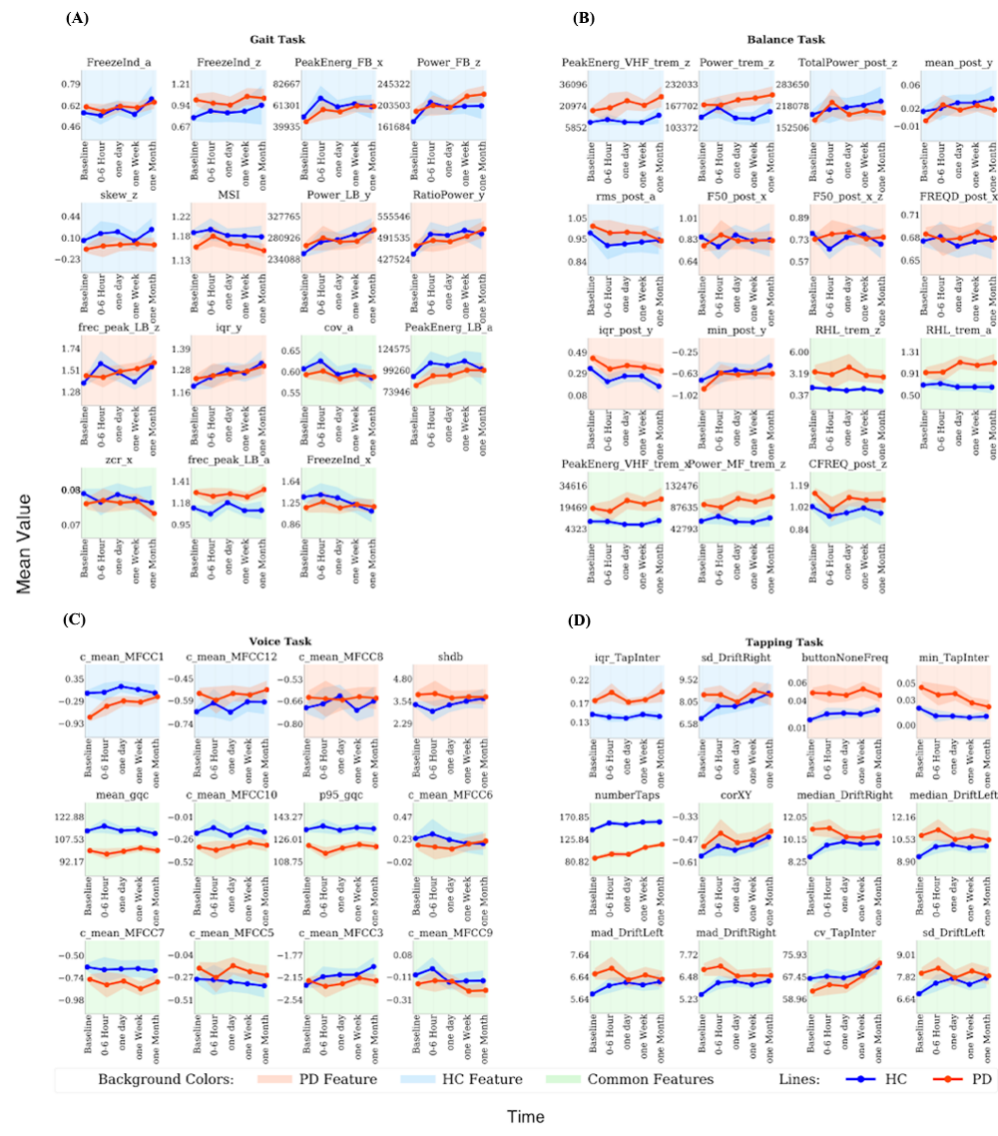
Mean value of the best performing baseline features across different time points, calculated for PD and HC separately. a: accelerometer average signal; HC: healthy controls; iqr: interquartile range; min: minimum value; PD: Parkinson disease; PeakEnerg: peak of energy; x: accelerometer mediolateral signal; y: accelerometer vertical signal; z: accelerometer anteroposterior signal. (A) Gait task. cov: coefficient of variation; FB: freezing band; frec_peak: frequency at the peak of energy; FreezeInd: freeze index; LB: locomotor band; MSI: mean stride interval; RatioPower: sum of the power in the freezing and locomotor band; skew: skewness; zcr: zero-crossing rate. (B) Balance task. buttonNoneFreq: frequency of tapping outside the button; CFREQ: centroidal frequency; F50: frequency containing 50% of total power; FRQD: frequency of dispersion of the power spectrum; HF: high frequency (>4 Hz); LF: low frequency (0.15-3.5 Hz); MF: medium frequency (4-7 Hz); post: postural; Power: energy between 3.5-15 Hz; RHL: ratio between power in high frequency and low frequency; rms: root mean square; TotalPower: energy between 15-3.5 Hz; trem: tremor; VHF: very high frequency (>7 Hz). (C) Voice task. c_mean: mean of the MFCC; gqc: glottis quotient close; log: energy of the signal and the first and second derivatives of the MFCC; MFCC: Mel-frequency cepstral coefficients; p95: 95th percentile; shbd: shimmer. (D) Tapping task. corXY: correlation of X and Y positions; cv: coefficient; DriftLeft: left drift; DriftRight: right drift; mad: median absolute deviation; numberTaps: number of taps; sd: standard deviation; TapInter: tap interval.

**Figure 5:**
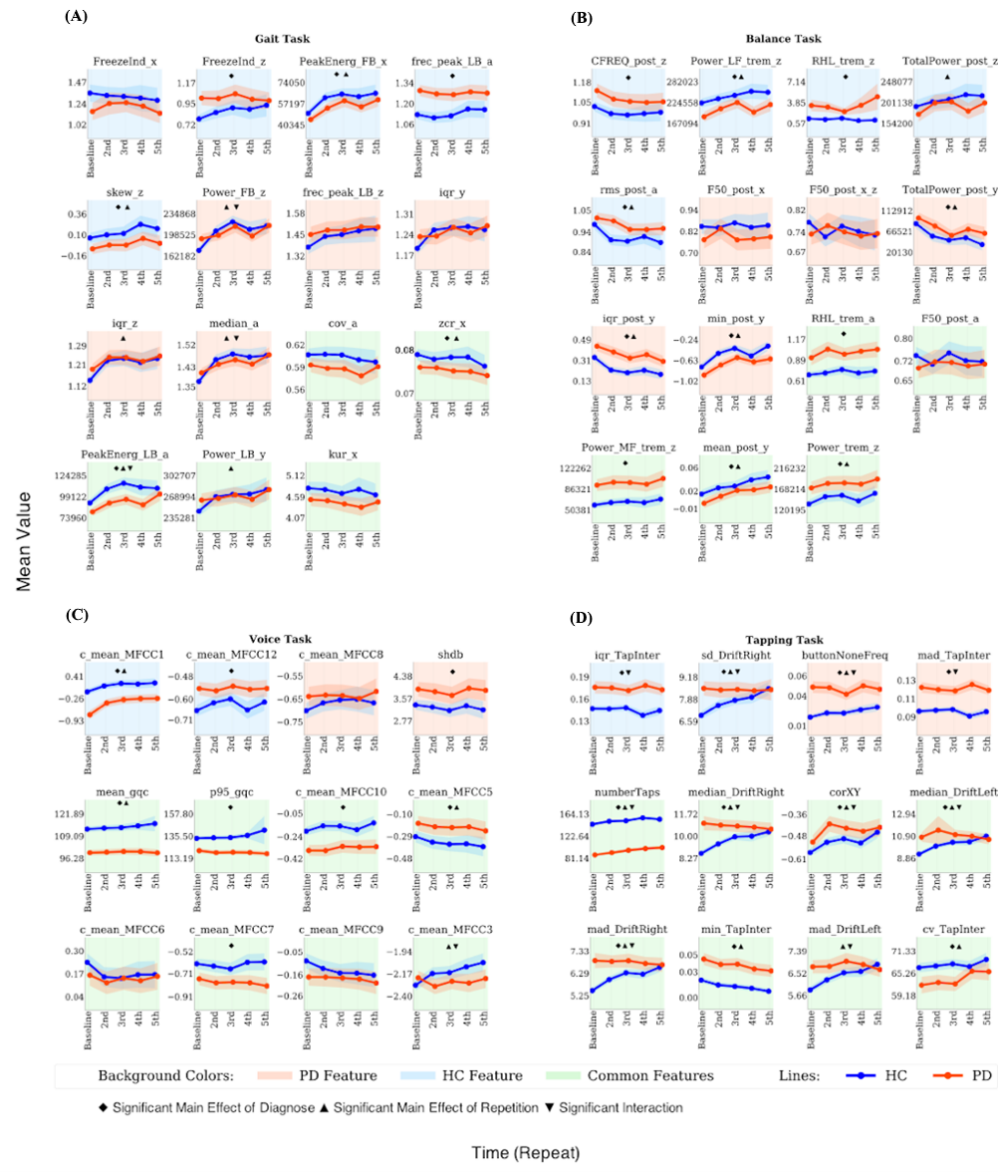
Mean value of the best performing baseline features across repetitions, calculated for PD and HC separately. a: accelerometer average signal; HC: healthy controls; iqr: interquartile range; min: minimum value; PD: Parkinson disease; PeakEnerg: peak of energy; x: accelerometer mediolateral signal; y: accelerometer vertical signal; z: accelerometer anteroposterior signal. (A) Gait task. cov: coefficient of variation; FB: freezing band; frec_peak: frequency at the peak of energy; FreezeInd: freeze index; kur: kurtosis; LB: locomotor band; MSI: mean stride interval; RatioPower: sum of the power in the freezing and locomotor band; skew: skewness; zcr: zero-crossing rate. (B) Balance task. buttonNoneFreq: frequency of tapping outside the button; CFREQ: centroidal frequency; F50: frequency containing 50% of total power; FRQD: frequency of dispersion of the power spectrum; HF: high frequency (>4 Hz); LF: low frequency (0.15-3.5 Hz); MF: medium frequency (4-7 Hz); post: postural; Power: energy between 3.5-15 Hz; RHL: ratio between power in high frequency and low frequency; rms: root mean square; TotalPower: energy between 15-3.5 Hz; trem: tremor; VHF: very high frequency (>7 Hz). (C) Voice task. c_mean: mean of the MFCC; gqc: glottis quotient close; log: energy of the signal and the first and second derivatives of the MFCC; MFCC: Mel-frequency cepstral coefficients; p95: 95th percentile; shbd: shimmer. (D) Tapping task. corXY: correlation of X and Y positions; cv: coefficient; DriftLeft: left drift; DriftRight: right drift; mad: median absolute deviation; numberTaps: number of taps; sd: standard deviation; TapInter: tap interval.

Next, we evaluated the longitudinal stability of these most reliable features. Using rm-ANOVA, we tested for the main effects of diagnosis, repetition (first, second, third, fourth, and fifth), and their interaction (Figures 4 and 5, Tables S9 and S10-S13 in Multimedia Appendix 1). A significant main effect of diagnosis across all time points was observed for 6 out of 15 gait features, 11 out of 15 balance features, 8 out of 12 voice features, and 11 out of 12 tapping features. A significant effect of repetition was found for 8 out of 15 gait features, 8 out of 15 balance features, 4 out of 12 voice features, and 10 out of 12 tapping features. A significant diagnosis-by-repetition interaction effect was identified for 3 out of 15 gait features, 0 out of 15 balance features, 3 out of 12 voice features, and 9 out of 12 tapping features. Further, we tested for the main effects of the elapsed time between repetitions and its interaction with diagnosis (Tables S18-S21 in Multimedia Appendix 1). A significant main effect of elapsed time was observed for 1 out of 15 gait features, 2 out of 15 balance features, 5 out of 12 voice features, and 5 out of 12 tapping features. A significant diagnosis-by-time interaction effect was observed only in 1 out of 15 balance features and 3 out of 12 tapping features.

In an additional sensitivity analysis, we further tested if the between-group differences and group-by-repetition interaction remain significant when controlling for age and sex. The results (Tables S14-S17 in Multimedia Appendix 1) show that a significant effect of diagnosis was still identified for 2 out of 6 gait features, 8 out of 11 balance features, 1 out of 8 voice features, and 10 out of 11 tapping features. A significant effect of repetition was still found for 6 out of 8 gait features, 7 out of 8 balance features, 3 out of 4 voice features, and 10 out of 10 tapping features. Also, a significant main effect of diagnosis-by-repetition was still observed for 1 out of 3 gait features, 1 out of 1 balance feature, and 8 out of 10 tapping features.

### Medication Effects 

Lastly, we tested which of the most reliable features identified above also display sensitivity to PD medication. For this we compared the conditions reported by the patients as being before PD medication, after PD medication, or at best. A significant effect of PD medication was only observed for 2 out of 15 gait features, 1 out of 15 balance features, 2 out of 12 voice features, and 1 out of 12 tapping features (Figure S3, Tables S9 and S10-S13, medication column, in Multimedia Appendix 1).

## Discussion

### Principal Findings

Here we assessed the longitudinal test-retest reliability and stability of DB measures related to gait, balance, voice, and finger dexterity impairments in PD. We found a wide range of test-retest reliabilities across tasks and features ranging from poor to excellent, with highest reliabilities observed for voice followed by the tapping task. Only a few features had medium to large effect sizes for differentiation between PD and HC. For all tasks, a substantial percentage of features displayed significant longitudinal alterations in their mean values over time.

Overall, tapping and voice tasks revealed a better performance compared to gait and balance tasks with respect to test-retest reliability and observed effect sizes. Balance and gait tasks displayed consistently poor test-retest reliabilities as well as low effect sizes for differentiation between PD and HC, calling into question their usability for home-based applications. In contrast, best performing voice features displayed fair to excellent test-retest reliabilities across repetitions but also over weeks and months.

Unlike some previous studies that showed good performance and moderate to excellent correlation of gait and balance features with clinical score [4,39], the overall poor performance of these tasks in the m-Power study may be explained by the nature of these tasks, which requires strict supervision and monitoring. Both may not be sufficiently achieved in the self-administered setting of the m-Power study. Overall, acceleration-related features in the gait task and tremor-related features and those selected from frequency domain in the balance task displayed the best performance for the respective task [23,40]. The features related to Mel-frequency cepstral coefficients for the voice task displayed the highest effect sizes for this task, which is in line with previous studies showing its ability in identifying pathological speech [41,42]. In line with previous studies, features related to intertapping interval and precision of the tapping task (eg, number of taps, taps drift) displayed the best performance among all [43,44].

Most features showed a decrease in test-retest reliability with longer periods of time. This may reflect a consequence of the repetition effects and the group-by-repetition interaction observed in the analyses of variance for a substantial proportion of the features. Features selected from the tapping task were less sensitive to the effect of age and sex compared to other tasks. Overall, the effects of age and sex were not significant for most of the features. The analysis of elapsed time between repetitions also revealed that the time difference between repetitions did not have a significant effect on most of the features. ICC values obtained from the PD and HC groups were largely similar, suggesting that other non-PD related sources of variation may have played a larger role in the observed low ICC values. Determining these reasons requires more controlled experiments than provided by the m-Power study.

Despite a significant difference at baseline, several features did not differentiate PD and HC when using data from all time points. This effect became most pronounced for the gait task, likely due to its poor test-retest reliability performance. Differential learning, variation in motivation, medication, reduced adherence to task instructions, and other physical and environmental parameters may contribute to this loss of differentiation [2,10,12]. While a clear differentiation of motivation versus learning effects on the often-abstract DB features is difficult in an observational study design, a possible way to provide inference on this issue is to compare the direction of alterations in PD and HC. Assuming that alterations in PD relative to HC reflect impairment, movement of a feature state toward PD is likely to reflect worsening due to reduced motivation, disease progression, or other similar factors. In contrast, movements toward HC is likely to reflect improvement and is therewith compatible with a learning effect. We find a mixture of both effects for most tasks, suggesting the presence of both aspects in DB longitudinal data. These observations are also in line with previous studies showing that training may reduce motor impairment in PD [45-47]. In particular, for the tapping task the difference between PD and HC disappears for several features, which is primarily due to a shift in performance in HC. These findings may point to a differential change in motivation across groups. While differential learning has been previously reported [45,48-52], the differential change in motivation is an important novel aspect to consider when comparing DB measures between PD patients and HC. Understanding the sources leading to this variability of DB measures over time is a vital and open question that needs to be systematically addressed to enable their application for specific clinical questions.

Most patients with PD take dopaminergic medication to alleviate their motor functions. However, the responsiveness to PD medication highly varies between patients. Besides good reliability and the ability to differentiate PD and HC, another important and desired quality of an effective DB is therefore to monitor PD medication response. Among the most reliable features from each task, only a few displayed significant but weak sensitivity to different medication conditions. One possible reason for this poor performance of DB measures in our study, as compared to some previous reports [20], might be the self-reported nature of the medication status in the m-Power data set, which likely introduced some noise variation (ie, different drugs and differences in time after administration). Nonetheless, our findings point to the need for further optimization of DB measures to increase their sensitivity to PD medication effects.

The self-administered design of the m-Power data set is also the major limitation of our study. In such an uncontrolled setting, accuracy in reporting the diagnosis and demographics, defining the medication status, and ensuring correct understanding of and compliance with the instructions may all have introduced variation into the study measures. The reported ballpark estimates for test-retest reliability and ability of the respective measures to differentiate between PD and HC therefore need to be carefully considered when interpreting our results. Another limitation of our study is the moderate adherence of participants in the m-Power study, which limited the number of participants who could be included in our analyses. Differences in age as well as lack of standardization of the time of day when the assessments were conducted are further sources of variation that may affect the generalizability of our findings [53]. Future studies may make inferences about the impact of different confounders such as comorbidities and disease severity on the longitudinal stability of DB. Also, further research is needed to establish the longitudinal stability of DB in the context of their relationship to clinical rating scales such as UPDRS.

Nonetheless, our findings clearly demonstrate the need for further optimization of DB tasks as well as for introducing careful monitoring and quality control procedures to enable integration of DB measures into clinically relevant applications.

### Data Availability

The m-Power data set used for this paper is available upon registration from Synapse [54].

## Appendix

Multimedia Appendix 1 Supplement.

## Abbreviations

DB: digital biomarkers
HC: healthy controls
ICC: intraclass correlation coefficients
PD: Parkinson disease
rm-ANOVA: repeated-measures analyses of variance
UPDRS: Unified Parkinson's Disease Rating Scale
